# Building capacity in mental health interventions in low resource countries: an apprenticeship model for training local providers

**DOI:** 10.1186/1752-4458-5-30

**Published:** 2011-11-18

**Authors:** Laura K Murray, Shannon Dorsey, Paul Bolton, Mark JD Jordans, Atif Rahman, Judith Bass, Helena Verdeli

**Affiliations:** 1 Department of International Health, Johns Hopkins University, Bloomberg School of Public Health, 615 N. Wolfe Street, 8th Floor, Baltimore, MD 21205, USA; 2 Dept. of Psychiatry and Behavioral Science, University of Washington, 2815 Eastlake Ave. E.; Suite 200, Seattle, WA 98102, USA; 3 Department of Research and Development, HealthNet TPO, Tolstraat 127, 1074 VJ, Amsterdam, The Netherlands; 4 School of Population, Community and Behavioural Sciences, Child Mental Health Unit, University of Liverpool, Mulberry House, Eaton Road, Liverpool L12 2AP, UK; 5 Department of Mental Health, Johns Hopkins Bloomberg School of Public Health, 624 N. Broadway, Baltimore, MD 21205, USA; 6 Department of Clinical Psychology, Columbia University, New York, New York, USA

## Abstract

**Background:**

Recent global mental health research suggests that mental health interventions can be adapted for use across cultures and in low resource environments. As evidence for the feasibility and effectiveness of certain specific interventions begins to accumulate, guidelines are needed for how to train, supervise, and ideally sustain mental health treatment delivery by local providers in low- and middle-income countries (LMIC).

**Model and case presentations:**

This paper presents an apprenticeship model for lay counselor training and supervision in mental health treatments in LMIC, developed and used by the authors in a range of mental health intervention studies conducted over the last decade in various low-resource settings. We describe the elements of this approach, the underlying logic, and provide examples drawn from our experiences working in 12 countries, with over 100 lay counselors.

**Evaluation:**

We review the challenges experienced with this model, and propose some possible solutions.

**Discussion:**

We describe and discuss how this model is consistent with, and draws on, the broader dissemination and implementation (DI) literature.

**Conclusion:**

In our experience, the apprenticeship model provides a useful framework for implementation of mental health interventions in LMIC. Our goal in this paper is to provide sufficient details about the apprenticeship model to guide other training efforts in mental health interventions.

## Background

Global mental health is an emerging priority in global health initiatives [1,2]. This body of research suggests that mental health counseling interventions can be adapted and implemented with positive outcomes in the area of mental health and functioning across cultures and in low resource environments [3-8]. The growth of research in this area is critical given the high burden of mental health disorders, which account for approximately one-third of YLD [Years Lived with Disability] among adults [2]. Depression, specifically, is the third leading global health threat. Despite the high prevalence and cost of mental health disorders, 90% of individuals with need do not receive treatment [9]. This is largely due to the scarcity of mental health professionals in LMIC, particularly in the lowest income countries and in rural/low-income regions within countries [10].

A range of randomized clinical trials testing mental health counseling interventions provided by local lay counselors, with little to no previous mental health training or experience, have demonstrated positive findings in the area of mental health, health, and functioning outcomes [11-13]. In Uganda, Interpersonal Psychotherapy (IPT) was effective in reducing the burden of depressive symptoms among adolescents living in internally-displaced persons camps [3] and adults in an area severely affected by the HIV epidemic [6,14]. In rural Pakistan, a CBT intervention for maternal depression was effective in both improving depressive symptoms and infant health (e.g., decreased diarrhea and improved immunization rates) [5]. In the studies where follow-up assessments were conducted (e.g., 6-months post-intervention completion), outcomes were maintained [5,15]. In a trial of a collaborative stepped-care intervention (i.e., MANAS) that included the provision of IPT for adult anxiety and depression in India, individuals who received the intervention were more likely than those who received enhanced usual care to: 1) recover at 6 months and 2) not meet the criteria for a diagnosable disorder [12].

Task shifting, employed in all these studies, involves moving the primary provision of the mental health intervention from mental health specialists (e.g., psychiatrists, psychologists, Master level providers) to lay counselors (i.e., limited to no mental health training or experience). This approach is responsive to the reality that addressing the mental health services gap requires an emphasis on a lay counselor workforce. Otherwise, scaling up mental health services for population-level impact is an unrealistic goal, given the limited number and unequal distribution of mental health specialists in LAMIC [16-18].

As evidence for the feasibility and effectiveness of specific interventions begins to accumulate, entities providing and supporting mental health interventions in LMIC (e.g., Ministries of Health, non-governmental organizations, community-based organizations) would benefit from guidelines on how to train and supervise these treatment skills to local providers. As stated in the recommendations from Mental Health Gap Action Programme (mhGAP), "pilot or experimental projects are of little value until they are scaled up to generate a larger policy and programme impact" [19]. Implementation guidelines for training and supervision of lay counselors, as part of task shifting, are an essential element of building mental health programs in LMIC.

The dissemination and implementation literature on building local capacity among lay (or any type of) counselors in low resource countries is relatively limited [for exceptions see [6,20-23]]. Published randomized trials of intervention outcomes [3,5,6,12,14,15] typically provide some detail on training and supervision. However, this information is limited in nature, due to a focus on reporting study procedures and outcomes, rarely sufficient for replication of the training and supervision approach. General guidelines do not currently exist and this gap in the literature is not well addressed in the recently launched mhGAP, due to its broader goals [19]. In the absence of guidelines, programs focused on mental health interventions or psychosocial support provided in LMIC typically consist of "one-off" or "train and hope" approaches (i.e., brief, one-time trainings, with limited pre- or post-training support).

Implementation science research, most of which has been conducted in the United States and other Western countries, clearly indicates that "one-off" training approaches may lead to initial knowledge change, but will not result in behavioral change in practice or counseling approach, even among mental health specialists [24-26]. Increasingly emerging in the implementation literature is the importance of supervision, coaching, and feedback in achieving fidelity, or adherence to the intervention [25-28]. As summarized in a recent paper on recommendations for training of mental health providers [29], "out of habit, we continue to conceptualize training and continuing professional education as a one-way broadcast from expert to trainee, primarily through didactic lecture, with only minimal feedback loops to learners from instructors regarding learning outcomes... for clinicians to become experts at a particular treatment, rather than achieve the minimal gains we tend to see, they must deliberately engage in target clinical behaviors, often and with feedback" (p. 8, para 3).

The goal of this paper is to elaborate on the training and supervision methods used in our collective work/studies in an attempt to more explicitly provide specific guidelines for lay counselor training and supervision in mental health counseling interventions in LMIC. The recommendations presented here were developed and employed over the last decade in varying low-resource environments, including Sri Lanka, Burundi, Indonesia, Sudan, Cambodia, Uganda, Zambia, Tanzania, Pakistan, Iraq, Nepal, and Thailand, with over 100 lay counselors. The authors are affiliated with different organizations and universities but are connected as scientific colleagues by a common interest in, and overlapping work on, improving outcomes for children, adolescents, and adults with unmet mental health needs in LMIC. In this paper, we have attempted to integrate our collective knowledge and experiences into a framework that can inform future training and supervision attempts.

Our focus is specifically on training and supervision of lay counselors to provide "Advanced Psychosocial Interventions" [18], defined by the mhGAP Intervention Guide as "interventions that take more than a few hours of a health-care provider's time to learn and typically more than a few hours to implement" (p. 4). We describe the elements of our approach, the underlying logic, and give examples drawn from our experiences. We also describe how this model is consistent with, and draws on, the broader dissemination and implementation (DI) literature. In doing so we hope to contribute to an emerging DI literature for LMIC, and ultimately, to the development of appropriate DI guidelines that can be adapted and used with a range of mental health interventions and populations in LMIC.

## Model and Case Presentations: An Apprenticeship Model for Building Local Capacity for Mental Health Services

### 1. Overview

In our work training lay counselors, the authors have used approaches that can be described collectively as an apprenticeship model. Our use of the term "apprenticeship" is based on similarities and overlaps between our approach to training and supervision of lay counselors and the basic approach by which apprentices are trained in many trades [30]. This overlap is listed below in Table 1 through Steps A-E, with associated components specific to the apprenticeship model for mental health counseling.

**Table 1:** Apprenticeship Models

Other Apprenticeship Models	Components specific to Apprenticeship in Mental Health Intervention Provision
A) Selection of apprentices who demonstrate interest and aptitude for the profession	▪ Selection of lay counselors ▪ Selection of supervisors
B) Coursework/training	▪ Initial on-site training in the intervention for counselors and supervisors ▪ Supervisor-specific training
C) Application of knowledge "on the job" under direct supervision and coaching	▪ Practice groups with local supervision and trainer consultation ▪ Supervision groups, close supervision of limited cases by local supervisor (and trainer consultation)
D) Ongoing expansion of knowledge and skills under supervision	▪ Supervisors: additional coaching on supervision techniques by trainers ▪ Counselors: Supervision groups for expanded number of cases ▪ Balancing fidelity and flexibility ▪ Supervisor monitoring of counselors' fidelity: self-report and observation of sessions.
E) Mutual problem solving by trainer and apprentice(s)	▪ Throughout all steps to account for cross-cultural nature.

**Figure 1:**
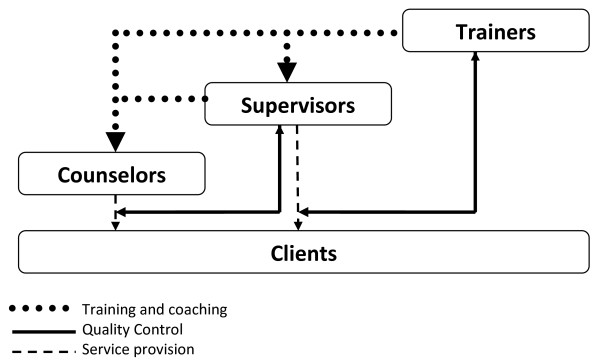
**Apprenticeship Model**.

The apprenticeship model used for mental health counseling in LMIC typically utilizes three main groups or individuals (as opposed to most trades with just a teacher and an apprentice): 1) Trainers - who are experts in the mental health intervention but usually from outside the project area, 2) Supervisors - who are ideally local individuals who have been chosen for a more advanced role, and 3) Counselors - local individuals who actually provide the mental health counseling intervention to clients. Figure 1 demonstrates the pathways of training and coaching, monitoring or quality control, and direct service provision, as well as highlights the interactional nature of the apprenticeship model across all three groups. These pathways overlap with Steps B-E above.

#### A. Selection of Apprentices

One of the first stages of the apprenticeship model involves identifying individuals from the local community, affiliated with local partnership organizations, who could be trained as lay counselors and supervisors. The criteria includes some basic indicators of aptitude and interest in holding a counselor or supervisor role, usually having at least a high-school equivalent education, strong interpersonal skills, and an interest and desire to learn how to help those with mental health disorders. Given the additional responsibilities of supervisors, we prefer that supervisors have some experience teaching and/or counseling. We have identified local supervisors both before and after the training (as counselors who show particular skill in the model). For instance, our work in some settings (e.g., Iraq, Uganda) has provided supervisors with more advanced skills (e.g., local psychiatrists or staff that were already supervising psychosocial programs) whereas in other settings, in which clinical specialists were not available (e.g., Zambia), supervisors were chosen from those who demonstrated more rapid and advanced uptake of the intervention skills during the in-person training. When possible, some authors have found that using a transparent, mutual selection interview process that includes a role-play that is objectively scored based on pre-determined criteria (e.g., ability to take and respond to coaching/feedback), as well as group discussion with candidates, can provide some protection against selection of individuals who may not be a good fit for a lay counselor or supervisor position.

Another aspect of provider selection includes consideration of available time. Many community members who meet these initial criteria may already have multiple responsibilities and roles in their organization or in the community, which is highly advantageous for socio-cultural understanding and access [23], but may create challenges around the time needed to apprentice. Therefore, to increase the responsibility of these individuals to take on mental health care provision, in our experience, other responsibilities have to be reduced to ease the burden on new counselors or supervisors. Organizational support of the apprentices is important, as the organization or community has to support the time commitment and resources needed (e.g., space to meet with clients, time allotted for training, supervision) in both the apprenticeship process (e.g., time for practice and supervision) and provision of the intervention [31-33]. When organizations are supportive, we have attempted to address sustainability and motivation by working with the local organizations and their staff to incorporate relevant incentives for participating counselors and supervisors. In some instances, these have included advanced training opportunities and certificates, or additional (e.g., bonus) monetary allowances for advanced skills. Some authors have used 'partnership contracts', a pre-training commitment and a job description (see Jordans & Sharma, 2004 [33] for details), which may help ensure that the investment of the training (both in human and financial resources) is not lost (e.g., organizations not using the newly trained counselors).

#### B. Coursework/Training

The initial training is a necessary, but not a sufficient stand-alone strategy, in building the basic skill-level of counselors [18,25]. In LMIC, the training should be conceptualized as the foundation for apprenticeship--the first step for learning and implementing a new intervention. For many trainees, the initial training is their first exposure to mental health and mental health interventions. While practice with clients under close supervision is the most critical element, the initial training provides the basic knowledge and skills. Research suggests that training efforts generally, and we believe particularly in LMIC, should be active, experiential, and incorporate various learning strategies [26,34]. The DI literature specifically highlights the importance of skills practice through behavioral rehearsal with coaching and feedback. Other active learning strategies, including small group work and trying out counseling strategies themselves (e.g., relaxation) also supplement purely didactic teaching. In most of our trainings, time allocated to practicing skills is greater than time spent in didactic training (e.g., lecture). For approximately every 30 minutes of didactics, about one hour of small-group, coached role-play practice follows. This focus on practicing skills is inline with the traditional trade apprenticeship model, in which learning is achieved by practicing new skills with oversight and coaching from an expert. Practice in the training initially involves coaching and feedback from the expert trainer; however, as the training progresses, the trainer encourages the local supervisors to take a more active role in coaching during the training.

Within an apprenticeship model, the trainer initially takes responsibility for guiding and teaching the main skills. For example, the trainer may first demonstrate the skill via a role-play or show a training video, then would answer questions directly. When counselors try the skill, the trainer would give significant guidance on the technique. As more trainees practice, the trainer shifts to asking questions to the trainees themselves to elicit answers and suggestions (e.g., "what is the next step that the counselor should take?"). This training technique, common in the adult learning literature [34], reflects the parallel therapy process in which the counselor will engage with clients: by teaching and then expecting greater initiative from clients over time.

##### Supervisor-Specific Activities in Initial Training

Research and our experience suggest that supervision is one of the most critical factors for effective implementation [25,26]. In our experience, the initial training provides an opportunity to begin building local supervisor capacity, versus an ongoing reliance on locally-based expatriates or outside experts. After the initial in-person training, the supervisor serves as the link between the trainer and the counselors (see Figure 1). As implementation of the intervention progresses, the supervisor takes on increasing independent responsibility for supervision and coaching.

Thus, supervisors are trained in the intervention *and *in supervision, either during the training (when supervisors are identified in advance) or subsequent to the training (when supervisors are identified during the training). In our experience, individuals who will provide a supervisory role need additional training, coaching, and support (i.e., apprenticeship from the trainer(s)) to learn *how *to supervise and how to begin setting up the ongoing supervisory and monitoring processes. This additional time can be used to cover a variety of supervision-specific topics including: a) reviewing components of the intervention and conducting more role-plays to ensure supervisor competency in the intervention; b) providing training on how to give constructive feedback on role-plays to improve counselors' skills; c) coaching on how to use questions to help counselors (rather than giving answers); and d) training on how to run a supervision group that is efficient and effective (e.g., setting agendas, guiding case presentations).

When supervisors are identified prior to the training, additional time can be set- aside during the training for only the supervisors. In this way, Step C of the apprenticeship model for supervisors -- application of knowledge "on the job" under direct supervision and coaching of trainers--begins during the initial training. We have found that this process also begins to transition the role of "teacher" or "expert" from the trainer to the local supervisors so that when the trainers leave, the counselors have already begun to recognize supervisor expertise (see Case Example below).

###### Case Example: A supervisor's increasing role in teaching, coaching, and leadership during am initial training

*A few days into the training, the trainers ask the supervisors to observe how the trainers teach, coach, and give feedback to the counselors while they are role-playing. As the training progresses, trainers begin to directly coach supervisors in role-plays, the trainer sits next to the local supervisor and provides coaching on when to interject and suggestions on what to say, but encourages the supervisor to deliver the actual supervision and feedback on the role-play. The trainers are then able to observe the supervisors' skills and provide feedback on their initial supervisory efforts*.

#### C) Application of Knowledge "On the Job" under Direct Supervision and Coaching

The goal of the initial in-person training is not only to provide training on the intervention, but also to set up mechanisms and structure for supporting intervention delivery post-training. Post-training support, in the form of supervision, is one of the strongest predictors of actual behavioral change [24]. Two recent reviews of mental health provider training, focused predominantly on US- and Europe-based efforts, conclude that, "ongoing supervision may be needed for actual therapist behavior change and skillful implementation" [25] (p. 3) and that, "there does not seem to be a substitute for expert consultation, supervision, and feedback for improving skills and increasing adoption" [26] (p. 462). The same appears to hold true in LMIC. In one of our studies of a Cognitive Behavioral intervention in Pakistan [23], the "supervision was the most valuable aspect of training" (p. 217). In our work training lay counselors in LMIC, specific supervisory support for counselors include practice and supervision groups that involve the supervisor apprenticing to the trainer, and then the counselor now apprenticing to the supervisor while "on the job." Apprenticeship efforts also include gradually increasing the local supervisors' responsibility for monitoring and quality assurance for the intervention.

##### Practice Groups with Local Supervision and Trainer Consultation

One strategy for facilitating apprenticeship at both of these levels (i.e., supervisor and counselor) involves having local supervisors run practice groups that begin immediately following the initial training (i.e., ideally within 1-2 weeks). Practice groups provide an opportunity for enhancing skills before counselors begin delivering the intervention to clients. In the practice groups, supervisors lead counselors in practicing components of the intervention, providing support and coaching. During this time, the supervisors are in weekly contact, typically via Skype, with the trainers to receive consultation and coaching themselves on providing supervision. Trainer-supervisor calls provide additional opportunities to enhance the skills of supervisors and allow the supervisors to serve as a conduit of information from counselors to trainers, and from trainers to counselors (see Figure 1).

In our work, practice groups have focused on additional role-plays of a particular intervention component or skill (e.g., relaxation skills, cognitive triangle) and solidification of skills learned in the training. Research shows that active, experiential learning strategies, like those used in the training, are an important supervision strategy [35]. Prior to providing the intervention to actual clients, in our experience, practice groups allow a period of "on the job" practice and receipt of coaching (trainers to supervisors, supervisors to counselors) that reinforces training and coursework from Step B of the apprenticeship model. We guide the supervisor to provide a review of the skill by asking questions of the counselors, and having the counselors practice the skill through a series of role-plays. Many of the authors have found it helpful for the trainers to provide clear suggestions of what the supervisor should be looking for in these role-plays (e.g., micro-skills, goals, potential challenges) to facilitate the supervisor's ability to effectively coach the counselors. In this way, these practice groups allow for additional "on the job" training with close direction and structure provided by the trainers through the local supervisors.

##### Additional Coaching on Supervision Techniques

As supervisors are beginning their supervisory duties, we have found it necessary to encourage the supervisor to provide *objective*(rather than subjective) reporting on what occurs in practice sessions. In other words, the trainers strive to "see a video" of what happened in the practice groups. For example, supervisors are coached to report how they introduced and explained a role-play and exactly what the counselors said within the role-play (vs. subjectively summarizing: "the role-play went well"). We have found it equally important to obtain objective reporting on supervisor feedback (e.g., what the supervisor is telling the counselors during the role-plays). Objective reporting allows the trainer to monitor counselor progression and to provide feedback and re-direction for counselors based on fact (rather than interpretations) that the supervisor can then relay to the counselor. Initially, the supervisor's feedback to counselors may predominantly consist of repeating the feedback from the trainer. Over time, however, the supervisor takes increasing responsibility for making his or her own suggestions about the counselor's practice and fidelity to the intervention. This type of reporting also permits trainers to track the supervisory techniques of the supervisors. Objective reporting of *what*the supervisor said and *how*the supervisor conveyed this information allows for additional coaching on supervisor skill in the intervention and feedback style (e.g., was positive feedback given first before constructive feedback? Did the supervisor model the correct way to do the skill?).

The authors use additional time on trainer-supervisor calls to enhance overarching supervisory skills (i.e., not specific to the intervention) such as setting agendas, how to coach counselors to give concise case presentations, and how to use questions to help counselors find answers themselves. Trainers also need to listen for time management skills. Local supervisors will eventually have to manage the review of multiple cases per counselor, and therefore their ability to efficiently and effectively set up role-plays, re-direct the group, and give concise feedback and suggestions is important. In our work, trainers may send agendas to help supervisors with the structure, time limits and topics for the first two or three practice groups, and then begin letting the supervisors structure the group themselves based on counselor need, with consultation from the trainers. These strategies are not new and significantly overlap with those used in other training approaches [36]. The difference here is that in the apprenticeship model, we use these strategies specifically to build local supervision capacity, in addition to building intervention-specific skills in counselors.

#### D) Ongoing Expansion of Knowledge and Skills under Supervision

##### Supervision Groups

Following a period of time dedicated to practice groups, each counselor and supervisor ideally obtains one or two "pilot" cases, so that close supervision can be provided prior to expanding the intervention to a large number of clients. We have typically required that supervisors take at least one pilot case themselves so that the trainer can provide supervision on the supervisor's delivery of the intervention (e.g., with the goal of ensuring adequate supervisor skill in the intervention). We have found that it is particularly helpful when supervisors begin a pilot case before the counselors so that they can be one to two weeks ahead of the counselors, and can anticipate challenges the counselors might encounter with their own clients.

By the time supervisors and counselors are beginning to provide the intervention to clients, the trainers would want to have adequate confidence in the supervisors' skills in the intervention (see the Limitations section for challenges, such as when the supervisor is not perceived to have adequate skills). Throughout the supervision of pilot cases, the supervisors continue to be in weekly contact with the trainers, as supervisors are increasingly apprenticing to become the local experts in the intervention. All cases are reviewed carefully, and feedback is provided on: 1) the supervisors' and counselors' implementation of the components of the intervention, and 2) on the supervisors' supervision skills (e.g., response and guidance of counselors). Supervisor objective reporting should be fairly solidified at this point and is increasingly important as counselors take on additional cases. Supervisors can now teach objective reporting to the counselors so supervisors can "see a video" of what is happening in a session with the client. The supervisors can then provide feedback to counselors in a similar, parallel fashion to how the trainers provide feedback to the supervisors. For example, in pilot cases, we find that the supervisor may tell the counselor exactly what to do and say in their next session, just as the trainer likely gave the supervisor very specific direction on how to coach counselor role-plays (see previous Case Example).

During the initial pilot cases, many supervisors are predominantly passing along the advice and suggestions of the trainer for future sessions. With additional pilot cases, supervisory skills continue to develop and the supervisors begin to give feedback more independently. For example, a counselor may report on challenges encountered when conducting cognitive restructuring with a client, and the supervisor responds on their own. The supervisor then describes to the trainer the feedback provided to this counselor Together, the trainer and supervisor would discuss how the supervisor's feedback might be inline with intervention fidelity and/or errors in the supervisors' response.

Towards the completion of pilot cases, ideally the supervisor and trainer(s) work together to assess the ability of the counselors to implement the intervention effectively, and their ability to take on additional cases. As the supervisors' skills advance, the trainer's role on calls shifts to asking questions of the supervisor in order to elicit his or her own conceptualization of counselor's cases. The trainer then confirms or suggests approaches for managing challenges or concerns identified by the supervisor.

###### Case Example: Supervisor's increasing independence

After completion of pilot cases in Southern Iraq, two supervisors identified counselor weaknesses, with the trainers, which were focused on two components of an intervention. The supervisors conducted "review sessions" independently on these components. They created the agenda themselves, directed the discussion, and responded to questions from the counselors. Supervisors reported back to the trainers.

##### Balancing Fidelity and Flexibility

Local supervisors and counselors play an important role in decisions about maintaining "flexibility within fidelity" [37] for intervention delivery, the critical line that balances room for creativity and adaptation to fit the population (or cross-cultural modifications) and fidelity (delivery of essential components).

###### Case Example: Supervisor balancing fidelity and flexibility

In Zambia, a counselor told a supervisor they were having difficulty with changing an unhelpful thought of "I am not worth anything." The supervisor asked for an objective report of what the counselor tried in session, and heard many statements about religious beliefs of the client. The supervisor suggested the counselor try asking, "What would God say about this thought?" The trainer and supervisor further discussed this on a call, and decided that this was in line with the intervention and also fit with the local population.

##### Supervision - Monitoring

Monitoring, or quality assurance, is important for both fidelity to the intervention, which is closely tied to positive outcomes [38,39], and for the timely identification of issues or challenges with the intervention that need attention in supervision. Therefore, we have provided training and support to supervisors in a structured way to monitor intervention fidelity as part of ongoing supervision. A variety of fidelity monitoring strategies is feasible in LMIC, including counselor self-report, live observation of sessions, and behavioral rehearsal (e.g., role-playing components as a proxy for observation). As described previously, behavioral rehearsal is significantly used through training and supervision in the apprenticeship model. At a minimum, the authors have trained counselors to complete self-report forms on what they did during the session: the component(s) delivered, any practice assignments outside of session, and monitoring of safety. The monitoring form is then used in supervision to identify challenges, such as a counselor spending multiple sessions on one component (e.g., relaxation) and not advancing to subsequent components.

In western contexts, counselor self-report has been found to have poor concordance with objective ratings [40]. Therefore, direct or indirect (via audiotape) observation, in which supervisors join or listen to a session to observe the counselor, is a more objective monitoring option. Although potentially the most accurate way to assess a counselor's skill level, it is often more difficult due to time involved and confidentiality issues. Live or taped observation can be challenging as some clients may experience discomfort with someone other than their counselor listening to, or joining the session. However, if presented as observation of the counselor, and not the client, this strategy is palatable and has been used effectively in a range of contexts. We have found that that direct observation can also be acceptable in LMIC. For example, it is common in Southern Iraq for counselors or supervisors to sit in on sessions, or even to join for the duration of the intervention, particularly when the counselor and client are of a different sex. Even when only conducted occasionally, live observation can significantly inform the supervisor about the counselor's skills and intervention implementation with clients.

Finally, counselors and supervisors are trained in ongoing monitoring of clients' symptoms. This is an important form of monitoring that has been identified as a recommendation for improving outcomes in mental health [41,42], with increasing attention internationally [43]. Ongoing symptom monitoring can be an important component of supervision, as it provides a means for regularly assessing client functioning and response to the intervention [44]. In many of our projects, we have included a short list of symptoms (e.g., approximately 10-15 items) that are administered during each session with the client. The supervisor reviews the symptoms with the counselor and makes recommendations based on results, and on the counselor's report of what happened in the session. For example, if a counselor is conducting gradual exposure with a client, and the monitoring form indicates high levels of avoidance symptoms, the supervisor may suggest another session of gradual exposure. The trainer and supervisor continue to review symptoms and examine which component of the intervention was delivered, to make a decision about when to move to the next component. This is also an example of Step E of the apprenticeship model, both adding to the knowledge base about how to make clinical decisions, and how to problem solve.

#### E) Mutual Problem Solving by Expert Trainer and Apprentice

Given the cross-cultural nature of implementing mental health counseling interventions in LMIC, the authors consider this step to cut across all steps of the apprenticeship model. Specifically, when a problem such as a particular belief (e.g., "I was possessed and thus needed to be raped"), or a safety issue (e.g., suicide plan) arises, it is important for the trainer to work closely with the local counselors and supervisors to jointly determine the best course of action. As summarized in Patel et al.'s [11] recent paper on lessons learned from implementing counseling interventions in LMIC, many components of counseling interventions developed in high-income countries are applicable cross-culturally; however, there are always some components that require adaptation to improve acceptability. Adaptation and modification involves joint discussion in which the trainer gives input specific to the treatment model, and the supervisors and counselors give input on the culturally appropriate approach and/or local resources.

##### Evaluation: Limitations and Challenges

As with any approach, the apprenticeship model in LMIC presents challenges that merit recognition, many of which, but not all, result from the reliance on local supervisors as the link between trainers and counselors. Challenges include: 1) supervisor attrition, 2) counselor attrition, 3) limited "experienced" capacity for handling clinical emergencies (e.g., concerns of suicide, homicide or child abuse), 4) time required, and 5) the need for the supervisors to speak the same language as the trainers.

Using an apprenticeship model, significant time is invested in training local supervisors, and these individuals have a high level of responsibility. Implementation can be threatened when a supervisor leaves a project unexpectedly, or is determined by the trainers to be somewhat ineffective in supervisor duties (e.g., limited intervention understanding, difficulty with managing supervision of multiple counselors with caseloads). It can be challenging to quickly identify and train a new supervisor, or shift greater responsibility to other supervisors, given the typically limited number of supervisors trained (e.g., usually 2-4). Some advance planning such as training additional "back-up" supervisors during the on-site training can be helpful, if possible, in planning for possible supervisor attrition. When the challenge is supervisor effectiveness, trainers have to decide whether to provide more intense consultation and coaching in an attempt to remediate the problem or, again, to either identify a new supervisor or shift responsibility to an existing supervisor. To identify supervisor effectiveness challenges early on, the authors have increased role-play evaluations and observations of "on the job" supervision, during the initial training, which can help screen out potentially under-skilled supervisors.

One specific challenge in LMIC related to supervisor attrition is that additional training sometimes makes individuals more marketable, thereby potentially increasing the likelihood of attrition with the training itself. As briefly discussed earlier, the authors have worked creatively with local organizations to identify incentives for supervisors to address retention (sometimes specified in a partnership contract).

###### Case Example: Effort to minimize supervisor attrition

In a recent implementation project in Thailand, some of the authors initiated a supervisor only group. This group meets twice a month, the local agency provides lunch, and the supervisors focus on one particular intervention component--sharing ideas, successes and challenges from their own groups. Our hope is that building cross-supervisor information sharing both raises the skill level of supervisors, and builds a supervisor cohort, that ideally prevents attrition by increasing peer support and interpersonal connections.

In summary, we believe that using local supervisors confers more benefits than challenges; however, our high reliance on these individuals necessitates some advance planning and creative brainstorming for each project.

Counselor attrition is also a challenge. The authors typically plan in advance for some attrition, and train a larger number of counselors knowing that some may not demonstrate competency in the intervention, and some may leave the project. During Step B, on-site coursework and training, as well as the frequent role-plays that make the training more active, serve to provide early identification of counselors that may not have the skills to implement the mental health intervention and/or have difficulty responding to coaching. Early identification of these individuals allows for potentially finding a different role for these individuals, such as recruitment or client retention, and can prevent additional time and resources being put into staff whose skills are better used in other areas. Additional protections against staff attrition may include organizational support, monetary compensation, adequate time allowance, and any opportunities for advancement in situations when strong counselors may be able to begin taking on some supervisory responsibilities as the counseling program grows.

When implementing mental health counseling in LMIC with lay counselors who have no formal mental health training, it is necessary to have a plan and support for clinical emergencies. When relying on lay counselors to deliver mental health interventions, it is necessary to have involvement from a local mental health specialist to help manage high-risk situations such as concerns about suicide, homicide, or child abuse. While trainers can provide some guidance, they are not on-site, and are not from the culture or local community. Therefore, in each location, an individualized safety plan is developed collaboratively between the trainers, local organization, and a local mental health specialist. In some countries in which we have worked, it is possible to have clinical specialists (e.g., physicians, psychiatrists or social workers) in the role of local supervisor.

###### Case Examples of Safety Plans

In a rare case in Southern Iraq, although the counselors had limited or no previous mental health training, all supervisors are either physicians or psychiatrists, who are available for emergencies and well-trained to handle them. In a project on the Thai border with displaced Burmese, one of four local supervisors is a physician, who is made available to field emergencies. In other areas (e.g., Zambia), the number of clinical specialists in the community limited our ability to identify supervisors with mental health expertise. In this situation, we linked with other groups and providers (e.g., a local hospital, psychology department in the university) in the area to ensure some access to a clinical specialist to handle emergencies, even if a clinical specialist was not in the supervisory role, and was not trained in the mental health intervention.

Any apprenticeship model of training is more time intensive than frequently employed "one-off" trainings. The authors do use shorter initial trainings, in line with recommendations for training lay counselors [11], but the ongoing supervision and consultation is time intensive. This requires time commitments and resources, which both can be challenging to find. We believe that in the long run, this model leads to greater cost-effectiveness by leaving more sustainable and quality mental health programs in place, given the growing body of research suggesting that interventions are not delivered with fidelity, or sustained without ongoing supervision and support [24-26].

Finally, in our experience so far, it has been necessary for the identified supervisors and trainers to speak the same language to enable weekly Skype consultation calls. Although initial trainings are often conducted with translators, we have not yet attempted to conduct the weekly consultation calls with translation. Since the majority of our trainers have English as a primary language, this has been a requirement for supervisors. This requirement limits the opportunity to a select few in some countries, and in other countries may pose a significant challenge. Ideally, over time these supervisors would be trained as national trainers to eliminate the language-related challenge.

## Discussion

This paper presents a model for lay counselor training and supervision in mental health interventions in LMIC that the authors have developed and used in various LMIC over the last decade. Given that dissemination and implementation of psychosocial and mental health interventions in LMIC is in the early stages, it is possible for these efforts to be informed by, and benefit from, the broader literature on how to most effectively disseminate and implement interventions. Although most of the research on dissemination and implementation (DI) has been conducted in the United States and other high-income countries [24], the findings and conclusions may be relevant to low-resource settings. The DI literature suggests that even in high-resource settings, *how *initial instruction is provided, and *what *post-instruction support is given (i.e., supervision) are important for adoption and implementation of new interventions [45-47].

Two recent reviews of mental health provider training, including studies of varying combinations of training elements (e.g., didactic or active workshops, web-based training, different doses of expert supervision), indicate that training efforts must include active learning (vs. passive, lecture-oriented training), attend to contextual factors such as organizational support, and include post-training supervision [25,26]. Notably, both reviews specifically highlight the importance of supervision. Implementation efforts that did not include some form of ongoing supervision resulted in unacceptable levels of clinician fidelity and competency [25,45]. In another recent study, the dose, or amount of supervision received (versus the training approach), was the most significant predictor of competence and fidelity post-training [48]. Research demonstrates that without ongoing support, interventions are not sustained over time, with significant attenuation of program availability even within two to four years [49-51]. This DI literature supports the use of an apprenticeship model, such as that described here, in that it focuses significant efforts on supervision of counselors and building supervisor capabilities.

## Conclusions and Future Directions

The authors have taken an apprenticeship approach to training lay counselors and supervisors, which we believe offers a number of benefits for local sustainability and the possibility of scaling-up interventions for broader reach and population impact - even given the existing challenges. Considering the broader implementation literature and our own experiences, supervision--and particularly on the job coaching and feedback--is clearly one of the most important factors for implementation with fidelity and for sustainability efforts. Our research, and that of our colleagues on mental health counseling interventions for common mental health disorders suggests that when the apprenticeship model is employed, mental health interventions delivered by lay counselors can have a positive impact on a range of important domains--including mental health, health, and functioning [5,12,14].

There is considerable research needed to further our understanding of how to implement mental health counseling interventions effectively in LMIC. It is clear that in addition to outcome data on effectiveness of mental health interventions, increased attention needs to be paid to the dissemination and implementation (DI) science of effectively bringing psychosocial and mental health interventions to LMIC. Research indicates that implementation activities, organizational, counselor, and even client characteristics influence uptake and implementation of evidence-based practices [25,52]. Thus, studies that examine processes used to train, supervise, and support counseling providers are needed in low-resource settings. Research on implementation --factors associated with acceptability, appropriateness, fidelity, and sustainability, among other implementation outcomes--is needed to guide decision-making of researchers, policy makers, funders, and stakeholders. Ideally, implementation questions can be embedded into effectiveness trials of mental health interventions, to answer both clinical effectiveness and implementation questions, in a single trial [53]. This paper focuses specifically on mental health counseling interventions for common mental health disorders, given the experience of the authors. More research and attention to these processes, both for mental health counseling and other interventions (e.g., medication management, collaborative interventions for severe neuropsychiatric disorders) is needed to supplement the growing body of western implementation findings, and to guide ongoing and future efforts in addressing the gap in mental health care in LMIC. With this research, and continued attention to how we train and supervise, the true "promise" of psychosocial and mental health interventions--improved health, mental health, and functioning--may be achieved.

## Competing interests

The authors declare that they have no competing interests.

## Authors' contributions

All authors have actively participated in apprenticeship model work in a LMIC. All authors made substantial contributions to this manuscript in drafting, revising and giving final approval. The three lead authors (LM, SD, PB) conceived the idea for the manuscript.
